# Grading diabetic retinopathy (DR) using the Scottish grading protocol

**Published:** 2015

**Authors:** Sonia Zachariah, William Wykes, David Yorston

**Affiliations:** Associate Specialist: Diabetic Retinopathy Screening Service, Glasgow, Scotland.; Consultant Ophthalmologist and Clinical Director: Diabetic Retinopathy Screening Service, Glasgow, Scotland.; Consultant Ophthalmologist: Tennent Institute of Ophthalmology, Gartnavel Hospital, Glasgow, UK. **dbyorston@btinternet.com**

Although traditionally the features of DR have been identified through direct ophthalmoscopy or slit lamp biomicroscopy, digital photography is more sensitive than direct ophthalmoscopy and is comparable to slit lamp examination by a trained observer.^1^

A digital fundus camera has the following advantages:

- Fast and convenient imaging of the retina by a photographer
- Storage, archiving, and transmission of the images
- Use of the images for quality assurance (that is, having them checked by another person) to ensure that no cases of retinopathy go undetected
- Ability to enhance images – magnification, red-free, enhanced contrast, etc.

When using the Scottish Grading Protocol^2^, just one retinal photograph is taken, which is centred on the fovea. The field must extend at least 2 disc diameters (DD) temporal to the fovea and 1DD nasal to the disc for adequate visualisation.

## Features of retinopathy

The signs of diabetic retinopathy are covered on page 65 and on pages 70–71. For DR screening, certain signs are more important than others.

**Blot haemorrhages** should be distinguished from **microaneurysms**, not just by their darker appearance but also by their size – the larger diameter of a blot haemorrhage should be equal in size to, or larger than, the diameter of the widest vein exiting from the optic disc.

**‘For DR screening, certain signs are more important than others’**

Chronic retinal oedema results in precipitation of yellow waxy deposits of lipid and protein known as **exudates.** When blot haemorrhages and exudates are visible within the macular area, they are considered markers for macular oedema.

Signs of retinal ischaemia include blot haemorrhages, venous beading and intra-retinal microvascular anomalies (IRMA). **Venous beading** is a subtle change in the calibre (thickness) of the second and third order retinal veins which gives them an irregular contour resembling a string of beads. **IRMA** look like new vessels; however they occur within areas of capillary occlusion and do not form vascular loops. Unusual vessels with loops therefore, should be treated as NV.

## Grading of DR

Most grading protocols are based on classification systems for DR which track the appearance and progression of disease (for example, the Early Treatment of Diabetic Retinopathy Study, or EDTRS, classification). Location (distance from fovea) is important when grading maculopathy. Visual acuity can be used as a marker for macular oedema, although it may be affected by other pathology such as cataracts or refractive error.

The Scottish Grading protocol grades the severity of retinopathy from R0 to R4 and of maculopathy as a separate grade from M0 to M2 (Table 1). R6 is a stand-alone grade for poor quality images which cannot be graded. If patients have technical failures at photography they must undergo further screening by slit lamp biomicroscopy.

**Figure 1.**
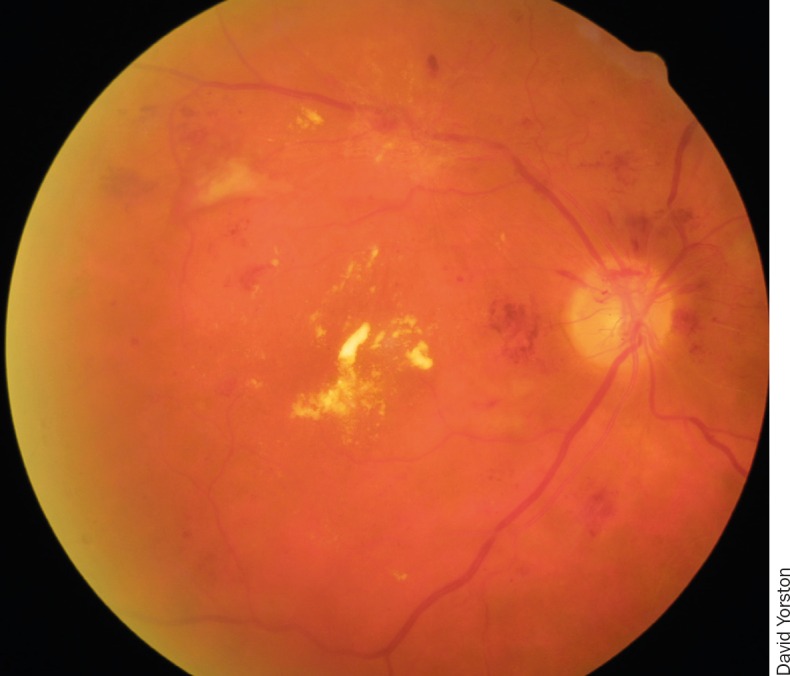
R3M2, The photograph shows multiple blot haemorrhages, corresponding to the R3 grade. In addition there are exudates within 1 disc diameter to the fovea, so the complete grade is R3M2

**Figure 2.**
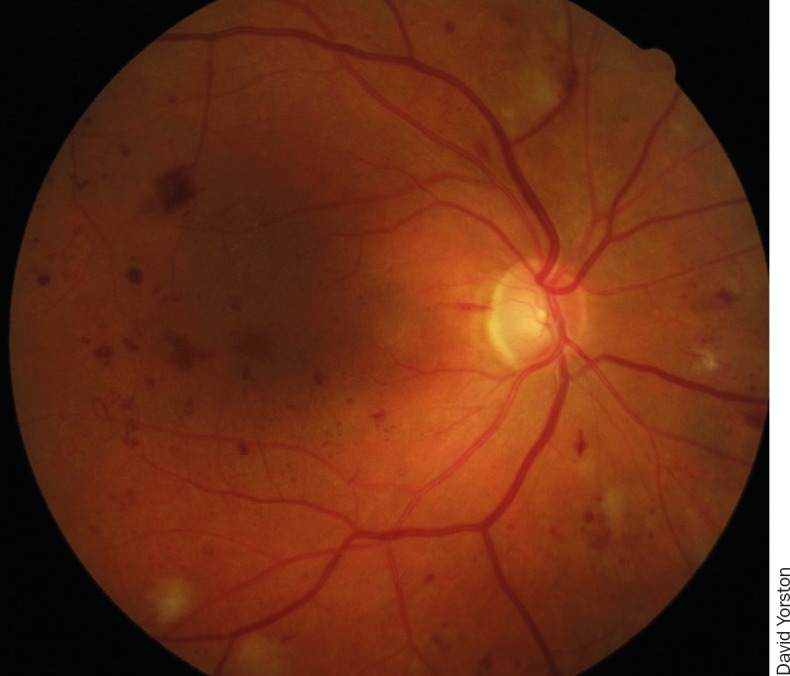
R3. There are blot haemorrhages and cotton wool spots. In addition there is a venous loop inferotemporal to the fovea. These features indicate severe ischaemia, corresponding to R3. There are no exudates visible

**Table 1.** The different grades of diabetic retinopathy (DR) in the Scottish Grading Protocol: features and outcomes

Grade	Features	Outcome
**R0**	No disease	Rescreen in 12 months
**R1**	**Mild background DR** Including microaneurysms, flame exudates, >4 blot haemorrhages in one or both hemifields, and/or cotton wool spots	Rescreen in 12 months
**R2**	**Moderate background DR** >4 blot haemorrhages in one hemifield	Rescreen in 6 months
**R3**	**Severe non-proliferative or pre-proliferative DR:** >4 blot haemorrhages in both hemifields, intra-retinal microvascular anomalies (IRMA), venous beading	Refer
**R4**	**Proliferative retinopathy** NVD, NVE, vitreous haemorrhage, retinal detachment	Refer
**M0**	No macular findings	12 month rescreening
**M1**	Hard exudates within 1–2 disc diameters of fovea	6 month rescreening
**M2**	Blot haemorrhage or hard exudates within 1 disc diameter of fovea	Refer
BDR – background diabetic retinopathy		
Hemifield – field of image divided by an imaginary line running across the disc and fovea		

When grading, the graders first assess the quality of an image on the basis of the clarity of the nerve fibre layer. Images considered of good enough quality are then inspected systematically, starting with the optic disc, then the macula and then all other areas. Using the red free filter is mandatory as it is essential to highlight subtle features such as microaneurysms and IRMA. Other tools such as the zoom and contrast enhancement are used to improve visualisation. A ruler is used to measure the size of blot haemorrhages and to measure the distance of exudates and blot haemorrhages from the fovea (in disc diameters) in order to set the maculopathy grade. Table 1 shows the different grades and their outcomes.^3^

## Conclusion

Screening has proved to be a vital tool in the fight against DR-related visual loss. An important measure of the successful implementation of screening is the reduced incidence of blindness due to sight-threatening diabetic retinopathy.^4^

**Figure 3a.**
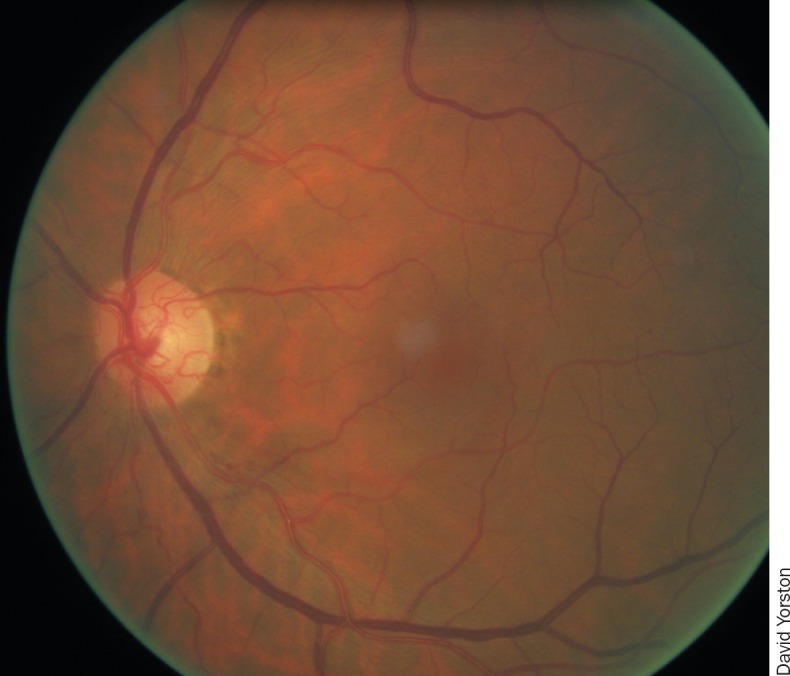
New vessels at the disc. There are new vessels at the optic disc, indicating high risk proliferative retinopathy. Note that there are few other signs of retinopathy, and you might miss the disc vessels if you are not looking for them

**Figure 3b.**
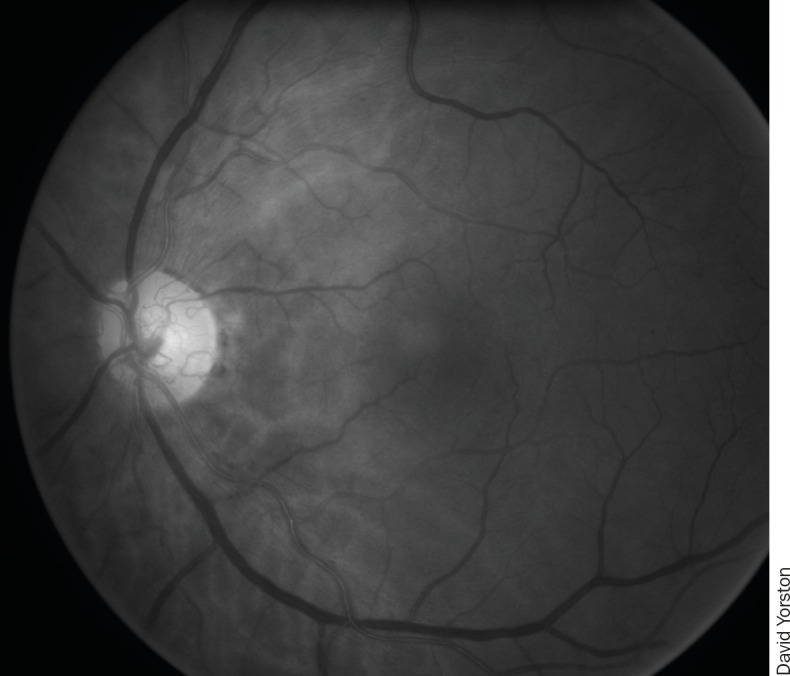
New vessels at the disc (red-free). The red-free version of this photo shows the new vessels at the optic disc more clearly. Altering the images, e.g. by using red-free, is a valuable tool for detecting retinopathy
